# Iliofemoral deep vein thrombosis after tibial plateau fracture fixation related to undiagnosed May-Thurner syndrome: a case report

**DOI:** 10.1186/1754-9493-7-12

**Published:** 2013-04-29

**Authors:** Niels A Foit, Qing-Min Chen, Blaze Cook, Eric Mark Hammerberg

**Affiliations:** 1 Department of Neurology, Universitaetsklinikum Freiburg, Breisacher Str. 64, D-79106, Freiburg, Germany; 2 Department of Orthopaedic Surgery, Denver Health Medical Center, 777 Bannock Street, MC 0188, Denver, CO 80204, USA; 3 Department of Radiology, Denver Health Medical Center, 777 Bannock Street, Denver, CO 80204, USA

## Abstract

**Background:**

May-Thurner Syndrome (MTS) represents an anatomic variation of the iliac vessels, in which the left common iliac vein is compressed by an overriding iliac artery. Patients with this abnormality are predisposed to the formation of a left-sided iliofemoral deep venous thrombosis (DVT). While DVT is a familiar complication in the setting of lower extremity trauma, there are no previous reports of MTS complicating the care of patients requiring orthopaedic surgery.

**Case presentation:**

We present the case of an extensive limb-threatening DVT in a patient with previously undiagnosed MTS, resulting after internal fixation of a left tibial plateau fracture. Four days after surgery, despite standard prophylactic anticoagulation, the patient developed an extensive occlusive DVT, extending from the common iliac vein to the popliteal vein. Successful diagnosis required a CT venogram in addition to standard lower extremity ultrasound exam. Severe lower extremity edema continued to worsen despite formal anticoagulation. Urgent mechanical thrombolysis was undertaken, followed by staged catheter-directed thrombolysis with recombinant tissue plasminogen activator (rTPA) and intraluminal stenting. Following this treatment, the patient was noted to have gradual but dramatic resolution of his lower extremity edema and swelling.

**Conclusion:**

The present case demonstrates the potential danger that may accompany MTS in the setting of lower extremity trauma. When an extensive left lower extremity DVT complicates the care of a patient with extremity trauma, clinicians should have a low threshold to pursue the diagnosis of MTS with advanced imaging studies. Venography remains the gold standard in diagnosis, but CT and MRI venography are less invasive and should allow for accurate diagnosis. In this case, formal anticoagulation proved to be ineffective, and endovascular intervention was required.

## Introduction

Deep vein thrombosis (DVT) is a well-known but feared complication following surgery to the lower extremities, especially in a prolonged trauma case [1,2]. Various causes of DVT are known and share a common mechanism, which involves the classic triad of Virchow: hemostasis, hypercoagulobility and endothelial injury [3]. Iliac vein compression syndrome (IVCS), also known as May-Thurner-Syndrome (MTS), is an anatomical variant which leads to venous obstruction and chronic venous insufficiency. The left common iliac vein is compressed between the right common iliac artery and the underlying vertebral body of the upper lumbar spine. It was first described by May and Thurner in 1957 and may present with acute and extensive DVT to the left lower extremity [4,5].

We detail the case of a DVT in an adult patient following open reduction and internal fixation (ORIF) of an unstable tibial plateau fracture, caused by previously undiagnosed MTS. We believe this to be the first report of MTS complicating orthopedic surgery.

## Case report

**Figure 1:**
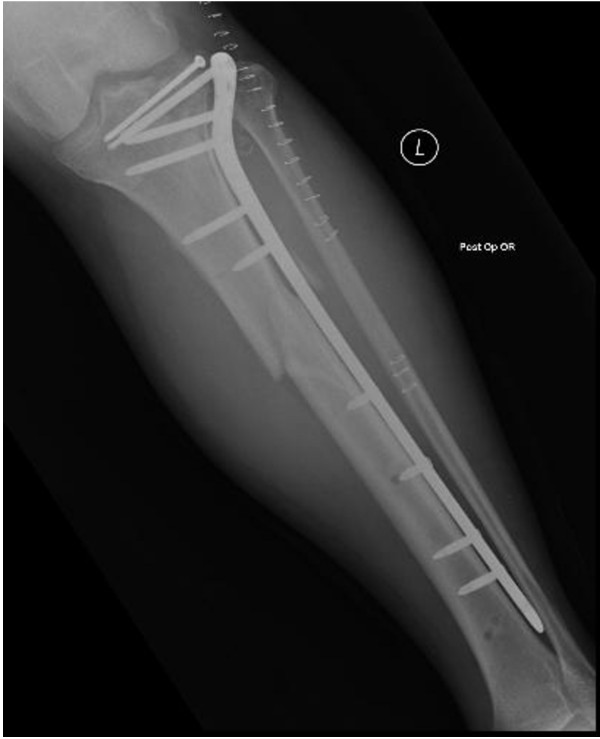
Postoperative radiograph following fixation of tibia fracture.

A 58-year old male was brought to our emergency department after a fall from a ladder. He sustained a bicondylar left tibial plateau fracture with an ipsilateral left diaphyseal tibia fracture. His BMI was 30.5, he had a 25-year history of smoking, and he took omeprazole for gastro-esophageal reflux disease (GERD). Exam demonstrated extensive soft tissue swelling to his left lower extremity. A temporary external fixator spanning both fractures was applied to allow soft tissue rest. The patient remained on the orthopaedic ward in stable condition and was brought back to the operating room for definitive ORIF ten days after initial treatment. Definitive management consisted of stabilizing the fractures with a 13-hole lateral locking plate (Synthes, West Chester, PA, Figure 1). DVT prophylaxis was performed by administration of subcutaneous heparin, 5000 units twice daily.

Four days after ORIF, the patient developed extensive swelling and erythema to the left lower extremity as well as frank scrotal edema. He was tachycardic and febrile. Compression gray scale ultrasound as well as Doppler imaging revealed an extensive occlusive DVT from the common femoral to the popliteal vein.

Systemic anticoagulation with heparin and warfarin was immediately started and a CT venogram was obtained, demonstrating an extension of the DVT into the common iliac vein. A stump of the left common iliac vein at the crossover from the right common iliac artery, diagnostic with MTS, was found. With still increasing soft tissue swelling and fear of a possible pulmonary embolism, a decision for an immediate endovascular intervention was made.

**Figure 2:**
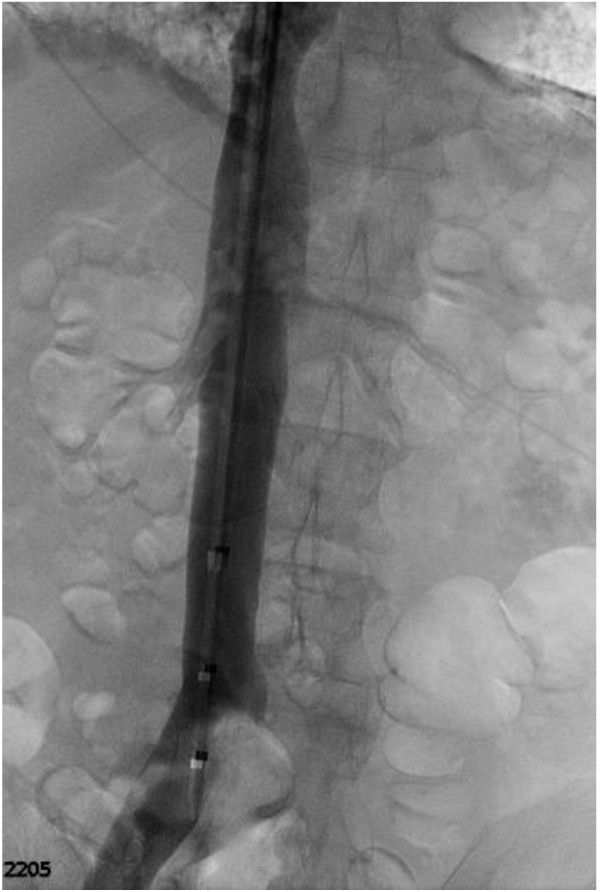
Venogram demonstrating occlusion of the left common iliac vein.

Both right internal jugular and right iliac vein were cannulated and a seven French inferior vena cave (IVC) sheath was positioned in the common iliac vein using a guide wire. Contrast venography was performed at this point and demonstrated the extensive DVT (Figure 2). A Bard-Ecclipse filter device (Bard Peripheral Vascular, Tempe, AZ) was deployed at the L1/L2-level. A glidewire and a glide catheter were then used to traverse the thrombus in the left common iliac vein. The glidewire was replaced by an Amplatz wire (Cook, Bloomington IN) over which an Angiojet (Possis, Minneapolis, MN) was advanced and mechanical thrombolysis was performed.

**Figure 3:**
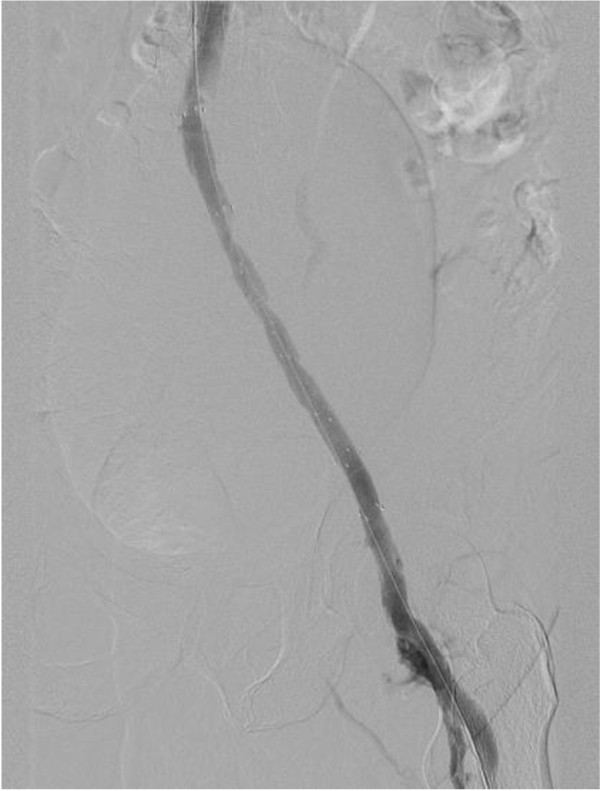
Venogram demonstrating restoration of venous outflow following angioplasty and stent placement.

The patient was closely monitored after the venous intervention, and formal anticoagulation was continued for two months. At this time, repeat ultrasound studies demonstrated extensive residual thrombus with extensive collateral formation. Catheter-directed thrombolysis with recombinant tissue plasminogen activator (rTPA) was performed, and stents were placed to maintain patency of the common iliac vein. Following 10 mm angioplasty of the stent segment, improved venous outflow was demonstrated by completion venogram (Figure 3).

Three months following thrombolysis and iliac stent placement, the patient was noted to have significant improvement in his lower extremity swelling. Following healing of his fracture, he resumed full weight-bearing on his left lower extremity.

## Discussion

Laterality in occurrence of DVT was first described by Virchow. He found the incidence of left-sided DVT to be 5 times higher than its right sided equivalent [6]. Nearly a century later, in 1956, an adequate explanation was found by May and Thurner [4]. They reported an anatomic variant of the iliac vessels, in which the left common iliac vein was compressed by an overriding common iliac artery of the right side. The chronic pulsating compression leads to endothelial changes and fibrosis in the intimal layer of the vein [7]. These lesions, called venous spurs by May and Thurner, represent the third component of Virchow’s triad. MTS is not a rare entity: In fact, it was present in 22% of the 430 investigated cadavers. A more recent study found even higher numbers [8]. Surprisingly; some studies suggest that only 1 to 3 out of 1000 patients with MTS will develop a DVT each year [5,9]. Usually, younger female patients between the ages of 18 and 30 are diagnosed with MTS, only 30% are male [10,11].

Diagnosis of MTS follows the general pathway of DVT diagnostics in the lower extremity. A clinical suspicion of a left or right lower extremity DVT should be investigated by obtaining duplex sonograms, although it is known to have limitations for iliac vein involvement. Presence of an iliofemoral thrombus should raise suspicion for MTS and further imaging should be used. Venography is still the diagnostic modality of choice, however CT as well as MRI venography are more easy to obtain and less invasive [12]. There are sporadic reports of a higher incidence of thrombophilia in MTS patients in the literature; therefore investigation should be carried out in conjunction with imaging [13].

Fractures of long bones distal to the hip put the patient in a high risk of developing a DVT. Some studies suggest that even without underlying anatomical abnormalities, the incidence of clinically occult DVTs following major trauma to the lower extremities is up to 28%, with up to 58% in polytraumatized patients [1,2].

In this case, a previously undiagnosed MTS, combined with recent trauma and prolonged immobility, could be identified as the cause for a DVT despite standard prophylaxis with heparin. This strikingly demonstrates the inefficiency of simple medical antithrombotic therapy in patients with MTS. A more aggressive therapy has to be chosen, involving endovascular techniques, to prevent post-thrombotic syndrome. The best outcome can be achieved by utilizing a combination of catheter directed thrombectomy, angioplasty and intraluminal stenting. Many authors recommend that an IVC filter device should be placed prior to venous interventions [14-16]. Acute thrombolysis with rTPA is a reasonable approach, but this may be contraindicated in the trauma setting due to the risk of hemorrhage. In a trauma case, mechanical lysis is the treatment of choice to immediately restore venous backflow in the affected limb. Stent angioplasty plays the most important role in long-term treatment [17-19]. Medical treatment includes oral anticoagulation for at least six months [20]. The patient should be electively brought back to the hospital for definitive endovascular treatment after fracture healing to minimize the risk of complications as well as to prevent further DVTs or chronic venous insufficiency [15-18].

## Conclusion

Patients with trauma to the lower extremities are in a high risk of developing DVTs. Young patients without other hypercoagulable risk factors who suddenly develop iliofemoral thrombosis should raise suspicion of an underlying MST, but it may even occur in older patients as in our case. Those individuals need a more aggressive workup and require early endovascular intervention to prevent recurring DVTs or chronic venous insufficiency. An interdisciplinary approach should be favored.

## Consent

Informed consent was obtained from the patient prior to publication. He consents to the use of his information for the purposes of publication of this case report.

## Competing interests

The authors declare that they have no competing interests.

## Authors’ contributions

NF performed the literature review and authored the first draft of the manuscript. QC collected patient data and helped with preparation of first draft. BC edited content of manuscript. EH supervised preparation of first draft of manuscript, revised manuscript, and prepared final draft. All authors read and approved the final manuscript.
